# Fabrication of Calix[4]arene Derivative Monolayers to Control Orientation of Antibody Immobilization

**DOI:** 10.3390/ijms15045496

**Published:** 2014-03-31

**Authors:** Hongxia Chen, Feng Liu, Fangjie Qi, Kwangnak Koh, Keming Wang

**Affiliations:** 1 Laboratory of Biosensing Technology, School of Life Sciences, Shanghai University, Shanghai 200444, China; E-Mails: hxchen@shu.edu.cn (H.C.); liufeng825@tom.com (F.L.); stefanieqfj@163.com (F.Q.); 2 Shanghai Key Laboratory of Bio-Energy Crops, School of Life Sciences, Shanghai University, Shanghai 200444, China; 3 College of Nanoscience and Nanotechnology, Pusan National University, Pusan 609-735, Korea; E-Mail: koh@pusan.ac.kr; 4 Department of Oncology, the Second Affiliated Hospital of Nanjing Medical University, Nanjing 210011, China

**Keywords:** calixarene, surface plasmon resonance (SPR), Immunoglobulin G (IgG), orientation, dipole moment

## Abstract

Three calix[4]arene (Cal-4) derivatives which separately contain ethylester (1), carboxylic acid (2), and crownether (3) at the lower rim with a common reactive thiol at the upper rim were synthesized and constructed to self-assembled monolayers (SAMs) on Au films. After spectroscopic characterization of the monolayers, surface coverage and orientation of antibody immobilized on the Cal-4 derivative SAMs were studied by surface plasmon resonance (SPR) technique. Experimental results revealed that the antibody could be immobilized on the Cal-4 derivatives spontaneously. The orientation of absorbed antibody on the Cal-4 derivative SAMs is related to the SAM’s dipole moment. The possible orientations of the antibody immobilized on the Cal-4 derivative 1 SAM are lying-on or side-on, while on the Cal-4 derivative 2 and Cal-4 derivative 3 head-on and end-on respectively. These experimental results demonstrate the surface dipole moment of Cal-4 derivative appears to be an important factor to antibody orientation. Cal-4 derivatives are useful in developing site direct protein chips.

## 1. Introduction

Immunosensors are among the most important diagnostic tools, which are widely used in medical diagnostics, forensic medicine, environmental analysis, and so on. Random and site-directed immobilization of antibody is used to made immunosensor. Random adsorption may suffer the influence of steric effects and surface density to reach a good immunoactivity object. In order to avoid random antibody immobilization and improve antigen binding site availability, site-directed immobilization methods are being constantly developed via, e.g., proteins binding the Fc region of immunoglobulins, using the antibody fragments and oxidized oligosaccharide moieties. However, these methods need affinity proteins, e.g., protein A, fusion proteins and advanced fabrication, which consume time and money. Also, researches show functional polymer and self-assembled small organic monolayers are used to immobilize antibody orientedly such as azopolymer, 16-mercaptohexadecanoic acid [1,2].

The calix[*n*]arenes are among the most widely studied organic macrocyclic host systems in supramolecular chemistry [3–6]. Their ease of selective chemical modification at either the para-aromatic position or the phenolic lower rim combined with the relative procedures for selective region functionalization is a key to their applications. Calixaren[4]arene (Cal-4) derivatives can be used as an artificial linker system for protein immobilization [7,8]. Han *et al*. [9,10], found that Cal-4 crown ether may have a unique surface chemistry allowing the formation of a self-assembled monolayer (SAM) on gold surface and tight binding of the captured proteins to the crown moiety of the linker molecules. The major binding force can be attributed to the ionized amine groups of the captured proteins, which bind to crown moiety of the linker molecule via host-guest interactions. Nevertheless, the mechanism of oriented adsorption of protein on the Cal-4 crown ether SAM is still not clear [11].

**Figure 1.**
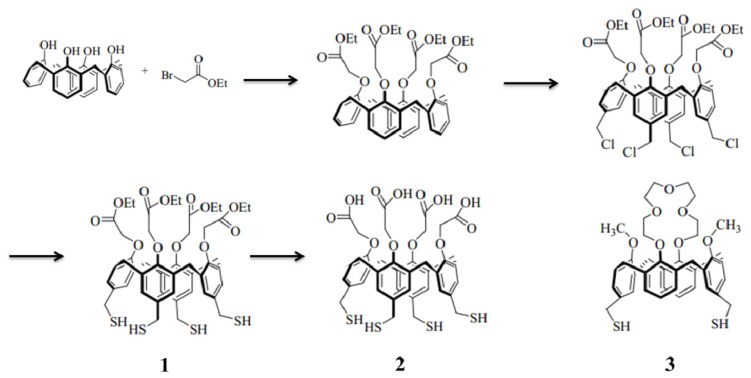
Molecular scheme of calix[4]arene (Cal-4) derivatives.

**Figure 2.**
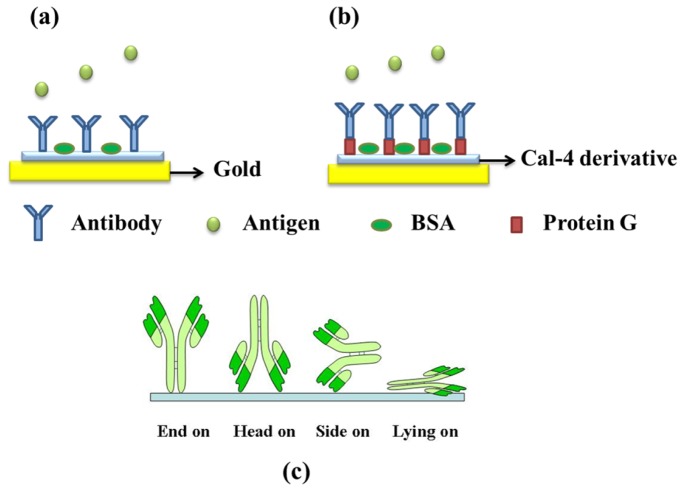
Schematic diagram of direct method (**a**); indirect method (**b**) and Ab orientations on the solid surface (**c**).

We synthesized and studied the immobilization of protein on several Cal-4 derivatives comparably. The structures are shown in Figure 1. Previous results showed that bovine serum albumin (BSA) can be immobilized on three derivatives in a monolayer with different surface coverage [8]. Also, we confirmed the controlled antibody orientation on the Cal-4 crown SAM by surface plasmon resonance (SPR) study [11]. However, how surface conditions of Cal-4 derivatives affect the orientation of adsorbed antibodies is not well known. In this study, we changed the function group of low rings at the Cal-4 to examine its effect on the orientation of immobilized antibody. For this purpose, three different Cal-4 derivatives separately containing ethylester (1), carboxylic acid (2) and crownether (3) at the lower rim were synthesized. As shown in Figure 2, Cal-4 derivatives were self assembled immobilized on gold chips through covalent biding between thiol group at the upper ring of Cal-4 derivatives and Au. Immunoglobulin G (IgG) was used as the model protein, since IgG is often employed as a molecular recognition element that binds specifically to its antibody with high affinity. The orientation of antibody can be determined by the interaction with its antigen. The immunoactivity was detected by SPR, which is a widely utilized technique for the study of molecular interactions [12–17]. Immobilization characteristics of Cal-4 derivatives and surface coverage of adsorbed protein on the molecular linker system were precisely measured by SPR. Orientation of the antibody on different Cal-4 derivative SAMs was simply and efficiently analyzed by SPR through calculation of immunoactivity. We estimated the orientation mechanism of the immobilized antibody on the Cal-4 derivatives by investigating the relationship of the orientation and the dominant factor leading to that orientation using computer simulation and SPR.

It was found that functional groups, such as –COOH, –COOEt and crown ether, greatly influenced the immobilization properties of Cal-4 derivatives. The crown ether Cal-4 derivative was very effective in promoting immunoactivity of immobilized antibody. The dipole moment of Cal-4 derivatives could be an important factor for antibody immobilization, which involved dipole-dipole interactions between the antibody and Cal-4 derivatives SAMs.

## 2. Results and Discussion

### 2.1. Characterization of Calixaren[4]arene (Cal-4) Derivatives Self-Assembled Monolayer (SAM) on the Gold Surface

**Figure 3.**
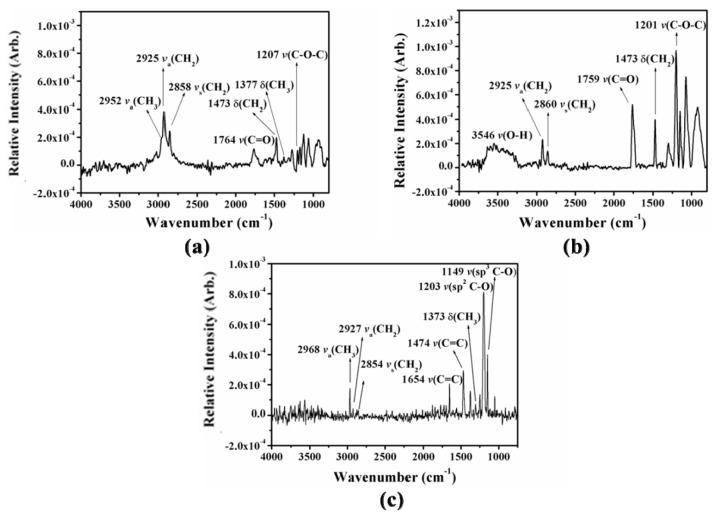
Fourier Transform Infrared (FTIR) spectra of (**a**) Cal-4 derivative 1 SAM; (**b**) Cal-4 derivative 2 SAM; and (**c**) Cal-4 derivative 3 SAM on gold surface.

The formation of each Cal-4 derivative SAM was characterized by Fourier Transform Infrared Reflection Spectroscopy (FTIR-RAS, Bruker Optics, Ettlingen, Germany), and Atomic Force Microscopy (AFM, Agilent 5500, Santa Clara, CA, USA). Figure 3 shows the FTIR-RAS spectra and assignment of the three Cal-4 derivative SAMs on Au surface. In the FTIR-RAS spectra confirm the typical absorption spectra of Cal-4 derivative SAMs on Au surface. In particular, the formation of Cal-4 derivative 2 SAM with carboxylic acid group from Cal-4 derivative 1 SAM is verified by appearance of ν(OH) stretching at 3546 cm^−1^ and absence of δ(CH_3_) stretching at 1377 cm^−1^ due to the elimination of ethylester group. In addition, after removing ethylester group of Cal-4 derivative 1 SAM, the peak intensity of νs(CH_2_) and νa(CH_2_) stretching mode is decreased in the FTIR-RAS spectra of Cal-4 derivative 2 SAM. These results effectively indicate the relative packing degree of each Cal-4 derivative SAM on the Au surface [18,19].

### 2.2. Characterization of Cal-4 Derivatives SAM on the Gold Surface

**Figure 4.**
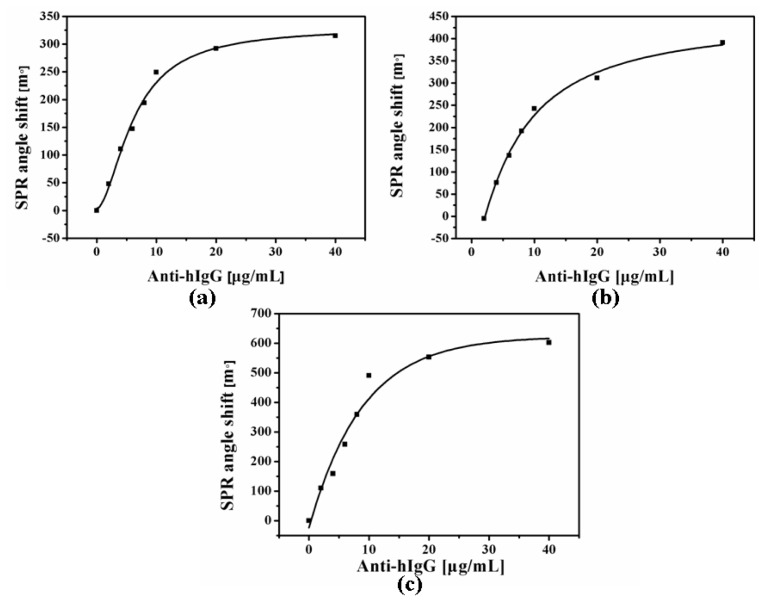
Anti-Immunoglobulin G (IgG) adsorption isotherm on the Cal**-**4 derivative 1 (**a**); Cal**-**4 derivative 2 (**b**) and Cal**-**4 derivative 3 (**c**).

For study of the antibody binding isotherm on the Cal-4 derivatives monolayer, nine different concentration solutions of antibody were prepared in the range of 2–40 μg/mL. Figure 4 shows the change of SPR angle with respect to antibody concentration immobilization on the three Cal-4 derivatives. As the adsorbed antibody molecule increased on Au surface, SPR angle shifts are gradually increased. However, the SPR increment is stabilized as the concentration of antibody increased in a range of over 10 μg/mL, which is a sufficient concentration for formation of antibody monomolecular film and antigen interaction. Thus, the increase of antibody on the Cal-4 derivative 2 is slower than that of derivative 1 and 3 (shown in Figure 4b).

### 2.3. Antibody Orientation on the Cal-4 Derivatives SAM

**Figure 5.**
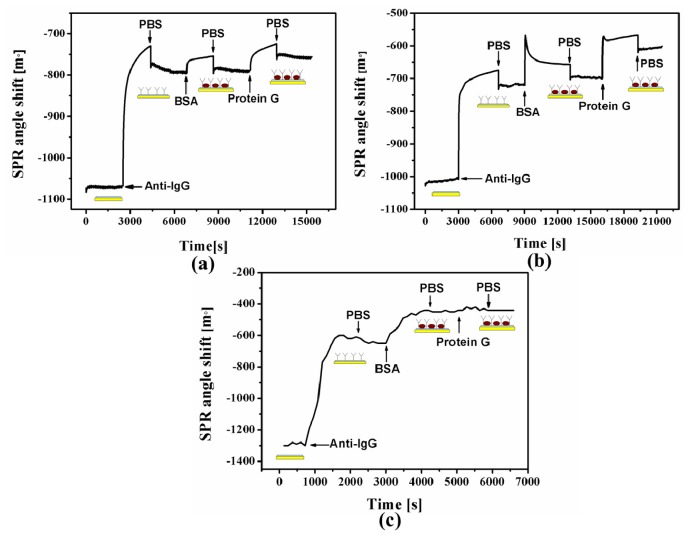
Surface plasmon resonance (SPR) angle shifts for the Cal-4 derivative 1 (**a**); Cal-4 derivative 2 (**b**) and Cal-4 derivative 3 (**c**) modified gold chip by sequential with anti-IgG, BSA and protein G.

Antibody has four possible orientations on a solid surface (Figure 2c): end-on (Fc closer to chip surface), head-on (Fab closer to the chip surface), side-on (Fc and one of the Fabs closer to the surface) and lying-on (Fc and two of the Fabs closer to the surface) orientations [20]. End-on or head-on orientation will lead to the same surface coverage and vertical orientation. For evaluation of the orientation property on the Cal-4 derivative SAMs, we designed a protein G–antibody interaction strategy and monitored by SPR. It is well known that protein G binds to the Fc region of the antibody [21]. Theoretically the antibody immobilized on the Cal-4 derivative SAMs with end-on orientation will have no interaction with protein G. As shown in Figure 5a,b, SPR response shows that antibodies are well immobilized on the Cal-4 derivative monolayers. After injecting of protein G, SPR angle shifts were observed as 32.6 and 94 m° for Cal-4 derivative 1 and 2 SAM modified surface respectively, which indicates part of immobilized anti-hIgG has a head-on or side-on orientations on sensor surface. However, following injection of protein G, no SPR angle shift was observed on Cal-4 derivative 3 modified surface (Figure 5c), which indicates no protein G bound on the antibody immobilized sensor surface. These data clearly demonstrate that antibody molecules immobilized on the Cal-4 derivative 3 surface form a tight molecular monolayer with end-on orientation, in such a way that the Fc domain of the antibody binds to the Cal-4 derivative 3 SAM surface hindering the protein G binding site on the Fc domain. In contrast, the Fab fragment of the antibody is open to the medium, allowing for free interaction of the antigen [22,23].

### 2.4. Immunoactivity of Antibody Immobilized on the Cal-4 Derivative SAMs

To confirm the orientation of Abs on the chip surface, we measured the chips’ immunoactivity further. Immunoactivity can be calculated from the molar ratio of antigen to antibody. Considering steric hindrance, the expected binding stoichiometry is 1:1 [7]:

(1) $${\text{Activity}}_{\text{Antibody}}={\text{Antigen}}_{\text{molecules}}/{\text{Antibody}}_{\text{molecules}}\times 100\%$$

Antigen_molecule_ and Antibody_molecule_ are molecules of bound antigen and coated antibody, respectively. Due to the linear relationship between SPR response (Δθ) and amount of protein coated to the chip surface, binding activity can be calculated [17]:

(2) $${\text{Activity}}_{\text{Antibody}}=(\Delta \theta_{\text{Antigen}}/\Delta \theta_{\text{Antibody}})\times ({\text{Mw}}_{\text{Antibody}}/{\text{Mw}}_{\text{Antigen}})\times 100\%$$

Δθ_Antibody_ and Δθ_Antigen_ are SPR angle shifts resulting from antibody immobilization and antigen binding with antibody, respectively. Mw_Antibody_ and Mw_Antigen_ are the molecular weight of antibody and antigen.

**Figure 6.**
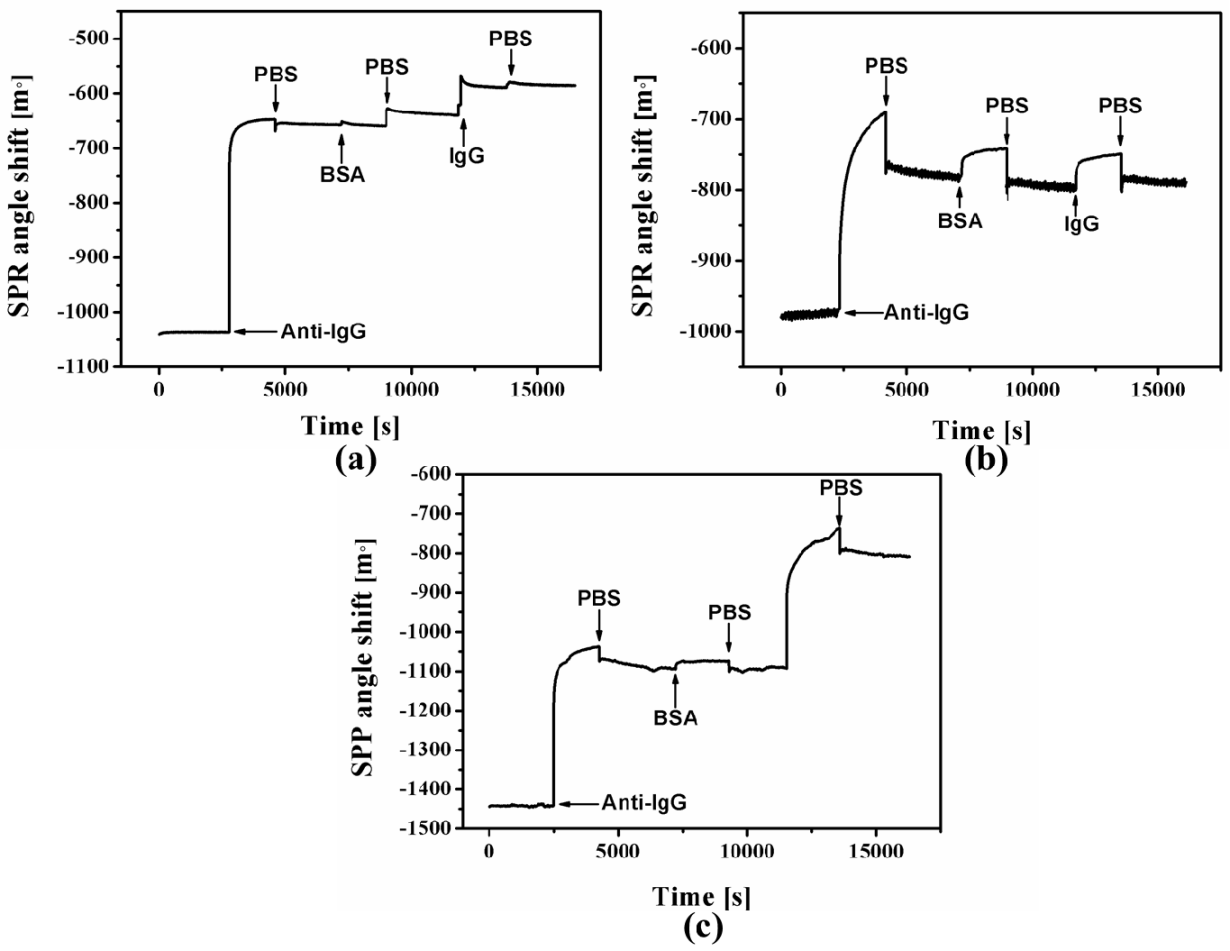
SPR angle shifts respect to the direct immobilization method: Cal-4 derivative 1 (**a**); Cal-4 derivative 2 (**b**) and Cal-4 derivative 3 (**c**).

Figure 6 shows sensograms with respect to antibody immobilization on Cal-4 derivative SAMs and then interaction with antigen. Figure 6a,c demonstrate that antigen can bind instantaneously with immobilized antibody on Cal-4 derivative 1 and 3. However, little SPR signal is observed on the Cal-4 derivative 2 modified surface (Figure 6b). Indirect immunoassay was also recorded by SPR and shown in Supplement (shown in Figure S1). Immunoactivity is calculated by the Equation (2). Calculation results are summarized in Table 1. In indirect methods of antibody immobilization to the surface of the Cal-4 derivative, SAMs are found to have similarities to theoretical boundaries of monolayer antibody coverage. Thus, in the direct immobilization strategy Cal-4 derivative 3 is comparable with the indirect strategy due to the antibody’s end-on orientation on the Cal-4 derivative 3 SAM. Among three Cal-4 derivatives, Ab immobilized on Cal-4 derivatives 2 SAM has the lowest immunoactivity. Considering the surface coverage and immunoactivity, the possible orientation of Anti-hIgG immobilized on the Cal-4 derivative 1 SAM is lying-on and side-on. The immobilized Anti-hIgG on the Cal-4 derivative 2 SAM may be head-on and side-on. Thus, Ab immobilized on the Cal-4 derivative 3 SAM shows a higher surface coverage and good immunoactivity due to its end-on orientation.

### 2.5. Mechanism of Ab Oriented on the Calixarene SAMs

**Figure 7.**
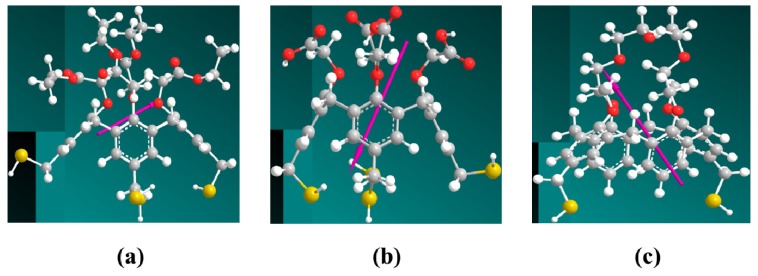
Dipole moments of Cal-4 derivatives simulated by ChemBio3D software: Cal-4 derivative 1 (**a**); Cal-4 derivative 2 (**b**) and Cal-4 derivative 3 (**c**).

It is generally found that the isoelectric point (IEP)—the net charge of a protein is zero at this pH value—of the (Fab)2 fragment is larger than that of whole antibody, while the IEP of the Fc fragment is smaller than that of the whole antibody. Therefore, the whole antibody molecule will have a dipole momentum pointing from Fc to (Fab)2 fragment, which is verified by the dipole calculation for all-atom antibody [24,25]. The all-atom dipoles of the three Cal-4 derivatives were simulated using Chem3D software (PerkinElmer Inc., Waltham, MA, USA). Dipole directions of Cal-4 derivatives are shown in Figure 7. According to the direction of the Cal-4 derivatives and antibody dipole, the immobilized antibody in a side-on or lying-on orientation can interact with Cal-4 derivative 1 SAM with a lower energy, while most Abs absorbed on Cal-4 derivative 2 and 3 in a head-on and end-on orientation respectively. These results indicate that the antibody can be immobilized on the Cal-4 derivatives spontaneously and oriented by the Cal-4 derivative’s dipole moment.

## 3. Experimental Section

### 3.1. Materials and Reagents

Protein G, hIgG, anti-hIgG (IgG2a), phosphate-buffer saline (PBS), tetrakis (chloromethyl) Cal-4, thiourea and Dimethyl Formamide (DMF) were obtained from Sigma (St. Louis, MO, USA). BSA was purchased from Zhaorui biotech Co. Ltd. (Shanghai, China). LiOH was purchased from Sinopharm Chemical Reagent Co. Ltd. (Shanghai, China). Cal-4 derivative 3 (crownether) was obtained from Proteogen Co. (ProlinkerTM, Seoul, Korea). Milli-Q grade (18.2 mΩ.cm^−1^) water (Barnstead, Billerica, MA, USA) was used for preparation of sample and buffer solutions.

### 3.2. Synthesis and Characterization of Cal-4 Derivatives

Cal-4 derivative 1 was synthesized according to the modified literature procedure [26]. Firstly, a mixture of tetrakis (chloromethyl) Cal-4 (216 mg, 0.22 mmol) and thiourea (200 mg, 2.6 mmol) was dissolved in DMF (15 mL). The mixture was then stirred at room temperature overnight under N_2_. After the solvent was removed by vacuum distillation at 100 °C, the residue was dissolved in 20 mL of 1 M-aquoues Na_2_CO_3_. Finally, the product was extracted with CH_2_Cl_2_ (20 mL × 3), The combined organic layer was dried over MgSO_4_, filtered, and concentrated to dryness to give glassy solid (200 mg, 94%). The product was characterized by ^1^H and ^13^C NMR (400 and 100 MHz, CD_3_OD, Bruker Co., Billerica, MA, USA). ^1^H NMR (400 MHz, DMF-d6) δ 6.72 (s, 8 H), 4.75 (overlapped with NH_2_), 4.67 (s, 8 H), 4.16 (s, 8 H), 4.12 (q, *J* = 6.6 Hz, 8 H), 3.20 (d, *J* = 11.5 Hz, 4 H), 1.20 (t, *J* = 6.6 Hz, 12 H); ^13^C NMR (100 MHz, CDCl_3_) δ 185.9, 172.7, 171.7, 157.7, 136.9, 131.1, 129.8, 73.0, 62.3, 36.8, 32.7, 15.0. Cal-4 derivative 2 (carboxylic acid) was converted from Cal-4 derivative 1 (ethylester) by treatment of 0.1 M LiOH for 3 h. The molecular schemes of Cal-4 derivatives are shown in Figure 1.

### 3.3. Formation of Cal-4 Derivative SAM

A fresh, bare gold chip (Echo Chemie, Utrecht, The Netherlands) was firstly treated with a piranha solution of 70% H_2_SO_4_–30% H_2_O_2_ (7:3, *v*/*v*) for 45 s, followed by rinsing with doubly distilled water until neutralized and dried with nitrogen flow gently. The gold chip was then used as a substrate for the formation of Cal-4 derivatives SAM. Cal-4 derivative 1 was prepared to 0.1 mM in chloroform for the formation of its SAM on Au surface [27,28]. The Cal-4 derivative 1 SAM was formed by immersion of the gold disk into the 0.1 mM Cal-4 derivative 1 overnight. Cal-4 derivative 2 SAM was converted from Cal-4 derivative 1 SAM through the hydrolysis of ethylene group by treatment of LiOH solution for 3 h. After the immobilization process, the chip was rinsed with chloroform and methanol, followed by being dried under N_2_ stream.

After rinsing with the same solvent of SAM formation process and drying under N_2_ gas stream, SAMs of Cal-4 derivatives were characterized by FTIR-RAS. The FTIR-RAS spectra were measured with a resolution of 2 cm^−1^. The glazing angle was maintained at 80° and a p-polarized IR beam was used as the light source.

### 3.4. Optimal Concentration of Antibody for Immobilization

SPR spectroscopic measurements were performed using an Autolab ESPRIT system at room temperature (Echo Chemie B.V., Utrecht, The Netherlands). For studying the optimal concentration of antibody for immobilization of the Cal-4 derivatives, antibody with 7 different concentrations in a range of 2–40 μg/mL in PBS solution (pH 7.4) was injected into the cell and detected by SPR. For blocking the nonspecific binding of protein G onto the site of Cal-4 derivative SAM without antibody, the chip was treated in 100 μL, 0.1 mg/mL BSA and followed by rinsing with PBS solution (pH 7.4). Lastly, 100 μL, 10 μg/mL of protein G in PBS solution was flooded onto the antibody layer, and followed by rinsing with PBS solution.

### 3.5. Antibody Interaction with Antigen

As shown in Figure 2a,b, two immunostrategies were used to study the immunoactivity of the antibody. In the case of the direct immobilization assay, the Cal-4 derivative SAMs modified chip was first stabilized by 0.01 M PBS solution; 100 μL, 10 μg/mL of Anti-hIgG was then injected into the cell for 30 min, followed by PBS rinsing to remove unbound antibodies. To block nonspecific binding sites, 0.1 mg/mL BSA was injected and incubated for 30 min, followed by PBS rinsing. Finally, 10 μg/mL of antigen in PBS solution was injected and incubated for 30 min, then rinsed with PBS buffer. In the case of the indirect immobilization assay using the protein G terminated antibody immobilization method, 10 μg/mL of protein G was first immobilized on the Cal-4 derivative SAMs and incubated for 30 min, followed by the steps used in the direct method.

## 4. Conclusions

In this study, three Cal-4 derivatives which contain ethylester, carboxylic acid and crownether as a functional group to interact with protein were immobilized on Au surface. Surface coverage and orientation of antibody immobilized on the Cal-4 derivative SAMs was studied by SPR. As a result, antibody can be immobilized on the Cal-4 derivatives spontaneously. The possible orientation of antibody immobilized on the Cal-4 derivative 1 SAM is lying-on or side-on, while the Cal-4 derivative 2 and Cal-4 derivative 3 head-on and end-on respectively. The change of the function group at the low ring of Cal-4 derivatives can affect the direction of dipole moments of Cal-4 derivative, which then results in the orientation of the immobilized antibody. Combined with Cal-4 derivatives’ easy modification at the phenolic lower rim, this method could be applied to site-specific immobilization of other biomolecules on the solid surface using on-demand artificial Cal-4 derivatives.

## Supplementary Information



## Figures and Tables

**Table 1.** SPR angle shifts according to Ab immobilization and interaction with Ag and Ab Immunoactivities on Cal-4 derivatives surface.

Layer& Immunoactivity	Cal-4 Derivative 1		Cal-4 Derivative 2		Cal-4 Derivative 3	
		
	Direct	Indirect	Direct	Indirect	Direct	Indirect
**Anti-IgG**	379.9	350.9	190.5	248.5	420	320
**IgG**	51.4	230.8	7.2	162.7	349.9	265.9
**Immunoactivity**	13.5%	65.8%	3.7%	65.5%	83.3%	83.1%
