# Supplementary material for: Fabrication of Calix[]arene Derivative Monolayers to Control Orientation of Antibody Immobilization
Source: Int J Mol Sci. 2014 Mar 31;15(4):5496–507. doi: 10.3390/ijms15045496 (PMC4013577; doi:10.3390/ijms15045496)
Supplement: Supplementary file 1 [file ijms-15-05496-s001.pdf]

Supplementary Information

Figure S1. Surface plasmon resonance (SPR) angle shift respect to the indirect immobilization method: Cal-4 derivative 1 (**a**); Cal-4 derivative 2 (**b**) and Cal-4 derivative 3 (**c**).

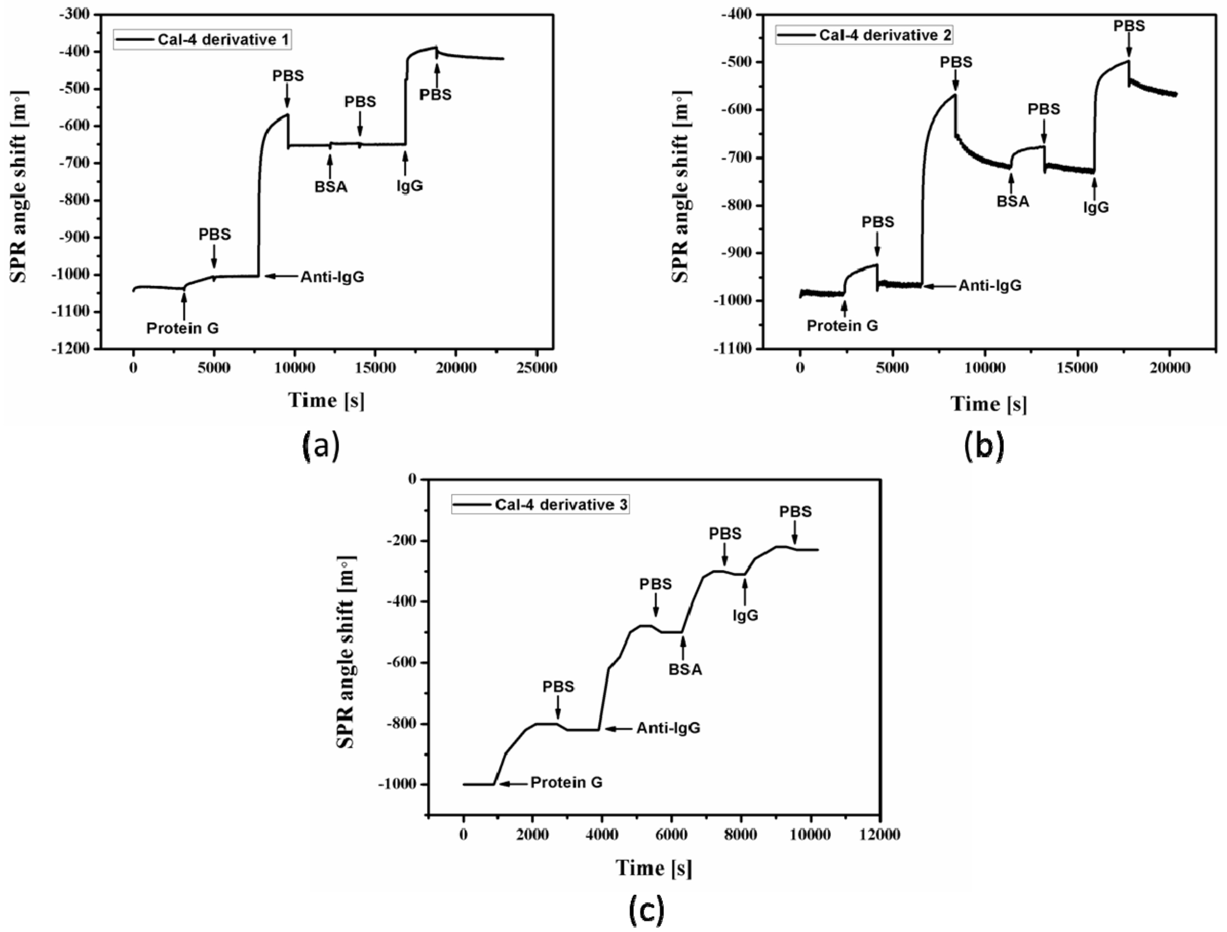

© 2014 by the authors; licensee MDPI, Basel, Switzerland. This article is an open access article distributed under the terms and conditions of the Creative Commons Attribution license (http://creativecommons.org/licenses/by/3.0/).
